# Basilar Artery Dolichosis Increases the Risk of Long–Term Recurrence in Patients With Pontine Infarction: A Prospective Cohort Study

**DOI:** 10.3389/fneur.2021.788145

**Published:** 2021-12-13

**Authors:** Shugang Cao, Xiaoxia Zhu, Qian Wu, Xiaoxing Ni, Jun He, Ping Cui, Tingting Ge, Jian Wang, Wen'an Xu, Mingwu Xia

**Affiliations:** ^1^Department of Neurology, The Second People's Hospital of Hefei, Hefei, China; ^2^Department of Geriatrics, The Second Affiliated Hospital of Anhui Medical University, Hefei, China; ^3^Department of Radiology, The Second People's Hospital of Hefei, Hefei, China

**Keywords:** brainstem infarction, stroke recurrence, vertebrobasilar dolichoectasia, basilar artery dolichoectasia, basilar artery dolichosis

## Abstract

**Background and Purpose:** Patients with basilar artery (BA) dolichosis are at high risk of acute pontine infarction (API), but the association between BA dolichosis and long–term stroke recurrence has received little attention. We aimed to identify the effect of BA dolichosis on the risk of long–term brainstem infarction recurrence in patients with API.

**Methods:** In this prospective cohort study, we enrolled 113 patients with API admitted to our department. BA dolichosis was diagnosed by a BA curve length >29.5 mm or bending length (BL) >10 mm on magnetic resonance angiography. The primary outcome was the occurrence of diffusion–weighted imaging (DWI)–confirmed brainstem infarction. The Cox proportional hazard model was used to detect possible predictors of brainstem infarction recurrence.

**Results:** Among 113 patients with API, 39 (34.5%) patients had BA dolichosis, and DWI–confirmed brainstem infarction recurred in 15 (13.3%) patients with a mean follow–up time of 31.2 months; the estimated 5–year incidence of brainstem infarction recurrence was 23.1% in patients with BA dolichosis, which was significantly higher than the incidence of 8.1% in patients without BA dolichosis. Cox proportional hazard analysis showed that age ≥65 years (hazard ratio (HR) = 3.341, 95% confidence interval (CI): 1.079–10.348, *P* = 0.036) and BA dolichosis (HR = 3.048, 95% CI: 1.069–8.693, *P* = 0.037) were significantly associated with a higher risk of brainstem infarction recurrence. In a subgroup analysis stratified by age, the patients aged ≥65 years with BA dolichosis had a higher risk of brainstem infarction recurrence (HR = 7.319, 95% CI: 1.525–35.123, *P* = 0.013).

**Conclusions:** This study indicates that BA dolichosis may increase the risk of long–term brainstem infarction recurrence in patients with API, especially in elderly patients, and therefore warrants more attention in clinical practice.

## Introduction

Basilar artery (BA) dolichosis refers to the elongation or curvature of the BA, with a BA curve length >29.5 mm or bending length (BL) >10 mm, and is one of the primary diagnostic criteria for basilar artery dolichoectasia (BADE) or vertebrobasilar dolichoectasia (VBD) (1). VBD/BADE is an uncommon vasculopathy that can easily lead to posterior circulation infarction, especially brainstem infarction (2–4). Kumral et al. (3) found that in 29 patients with posterior circulation infarction complicated with VBD, more than half of the infarcts were located in the pons. However, many patients with simple BA dolichosis cannot be classified as having VBD (5, 6). In the study by Del Brutto et al. (7) the proportion of BA dolichosis reached as high as 11.6% in community–dwelling older adults. The incidence of BA dolichosis is higher in patients with pontine infarction. The cross–sectional study by Kwon et al. (8) found that in the Korean population, the detection rate of BA dolichosis was 19.8% in patients with acute pontine infarction (API).

Based on the current quantitative criterion for BA ectasia (BA diameter >4.5 mm) (1), our preliminary study found very few patients (only one out of 101) with BA ectasia in the Han Chinese population. However, the detection rate of BA dolichosis was 33.6%. Further analysis showed that patients with BA dolichosis had larger infarct diameters at the maximal level of pontine infarction, and BA dolichosis was correlated with unfavorable 90–day outcomes (5), which may be attributed to persistent hemodynamic abnormalities and perforating artery disease due to BA dolichosis and/or bending. Therefore, it remains to be determined whether BA dolichosis or BA ectasia is more likely to cause posterior circulation infarction. Woltesr et al. (9) conducted a meta–analysis of 375 VBD patients from nine cohorts and found that the estimated 5–year incidence of posterior circulation stroke was 17.6%, which was higher than that of other clinical manifestations, such as cerebral hemorrhage and cranial nerve compression. Patients with BA dolichosis are prone to perforator artery disease of the BA, but the effect of BA dolichosis on long–term stroke recurrence is unknown. We conducted a prospective cohort study to investigate the effect of BA dolichosis on the risk of long–term recurrence in patients with API, with diffusion–weighted imaging (DWI)–confirmed brainstem infarction as the endpoint event.

## Methods

### Study Population

The study population consisted of patients with API in the Department of Neurology of our hospital. They were from an ongoing prospective cohort study for purposively determining the relationship between BA morphology and outcomes in patients with posterior circulation infarction in a single–center Han population. Patients with API were consecutively enrolled between July 2015 and June 2019 if they met the following eligibility criteria: aged 18–80 years, admitted within 7 days after onset, and diagnosed with API by DWI. Patients with a previous history of atrial fibrillation or newly diagnosed atrial fibrillation, segmental thickening of the BA or BA aneurysms (10), evidence of hemodynamically severe BA stenosis (≥70%) or occlusion affecting our measurements, or incomplete clinical or imaging information were excluded. This study was approved by the Institutional Review Board of the Second People's Hospital of Hefei. All patients provided written informed consent and were informed about the long–term follow–up plan of the study. The data supporting the findings of this study can be obtained from the corresponding author on reasonable request.

### Clinical Information and Assessment

Baseline clinical information were collected in detail on enrollment, including current smoking, drinking, hypertension, diabetes mellitus, and dyslipidemia, as well as systolic and diastolic blood pressure on admission. Hypertension was defined as a resting systolic/diastolic blood pressure of ≥140/≥90 mmHg on repeated measurements or taking antihypertension drugs. Diabetes mellitus was diagnosed when the patient had a fasting blood glucose ≥7.0 mmol/L or had taken oral hypoglycemic agents or been treated with insulin. Dyslipidemia was diagnosed if the patients had total cholesterol ≥5.60 mmol/L, triglycerides ≥1.81 mmol/L, or low–density lipoprotein ≥3.57 mmol/L or had taken antilipemic medication for these conditions. According to the etiology classification of API proposed by Kumral et al. (11), the stroke etiology was classified into vertebrobasilar large–artery disease (LAD), small–artery disease (SAD), basilar artery branch disease (BABD), and other and undetermined etiologies. The etiology of potential cardiac sources of embolism was excluded. Discharge medications, including antiplatelet agents, statin therapy, antihypertensive agents, and glucose–lowering agents, were also recorded in detail.

### Imaging Protocol and Analysis

All patients underwent conventional magnetic resonance imaging (MRI) with a 1.5–tesla MRI unit (Siemens Healthineers, Model: Avanto I class, Germany). The DWI scan parameters included a repetition time/echo time of 3,400/102 ms, a slice thickness of 5 mm, a dispersion mode of 3–scan trace, and a b–value of 0–1000. The location of pontine infarction on DWI was divided into pontine base infarction (extending to the basal surface of the pons) and deep pontine infarction (not reaching the basal surface). We conducted magnetic resonance angiography (MRA) using a three–dimensional time–of–flight (TOF) sequence with a repetition time of 25 ms, an echo time of 7 ms, and a slice thickness of 0.6 mm. Maximum–intensity projection reconstructed images were used for analysis. The evaluation method of BA features, mainly including BA diameter, BA curve length, BA length (BAL), and BL, was described in detail in our previous study (5). A BA curve length >29.5 mm or BL > 10 mm is diagnosed as BA dolichosis We defined a BA diameter >4.5 mm at any location along its course as BA ectasia. Patients meeting both of the above criteria were considered to have BADE (1). The line of BAL is used to determine the location of a BA curvature (toward the right or left side or straight) (12). The severity of the BA curvature was classified into no curvature (straight), moderate curvature (BL ≦ 10 mm) or prominent curvature (BL > 10 mm, namely BA dolichosis). We further evaluated the maximum bend of the BA (proximal, middle, or distal). BA hypoplasia (BAH) refers to a continuous diameter reduction all over the artery and a basilar artery diameter <2 mm (13). All vascular images were analyzed by two experienced neuroradiologists, and the mean values were recorded as the result for analysis. When there was any disagreement, a radiologist with 10 years of experience was consulted to resolve the issue. A Bland–Altman plot was used to analyze the agreement between two readers.

### Follow–Up Data

The enrolled patients with API were followed up by telephone interviews or outpatient visits every 6 months. Once the patients had stroke symptoms, they were hospitalized and submitted to complete DWI–MRI examination to determine whether there was acute cerebral infarction recurrence. The primary outcome was the occurrence of DWI–confirmed acute brainstem infarction. The endpoint date for follow–up was June 30, 2020. The follow–up interval was defined as the time between the stroke onset and recurrence, loss to follow–up, or death.

### Statistical Analysis

All statistical analyses were performed with SPSS 22.0 package for Windows (SPSS Inc., Chicago, IL, USA). Continuous variables were tested for normality by the Kolmogorov–Smirnov test and are expressed as the mean ± standard deviation (SD) or as the median (M) and interquartile range (IQR). Categorical variables are presented as proportions (%). The age of onset and BA diameter (normally distributed) were compared by Student's *t-*test. A chi–squared test or Fisher's exact test was used to compare the distribution of categorical variables between groups, including age grouped by median, sex, risk factors, medications at discharge, basilar artery features, location of pontine infarction, and stroke etiology. The time to the first recurrence of brainstem infarction was estimated by the Kaplan–Meier method and was compared using a two–sided log–rank test. The association of brainstem infarction recurrence with potential factors was assessed by the Cox proportional hazard model, with and without adjustment for the prespecified factors mentioned above. Subgroup analysis was also performed when necessary. Hazard ratios (HRs) and 95% confidence intervals (CIs) were subsequently calculated. All tests used a two–sided *P* value of 0.05 as the threshold for statistical significance. All plots were drawn in R version 3.6.1 with the survival (version 3.2.1) and survminer (version 0.4.6) packages.

## Results

### Clinical Data

**Figure 1:**
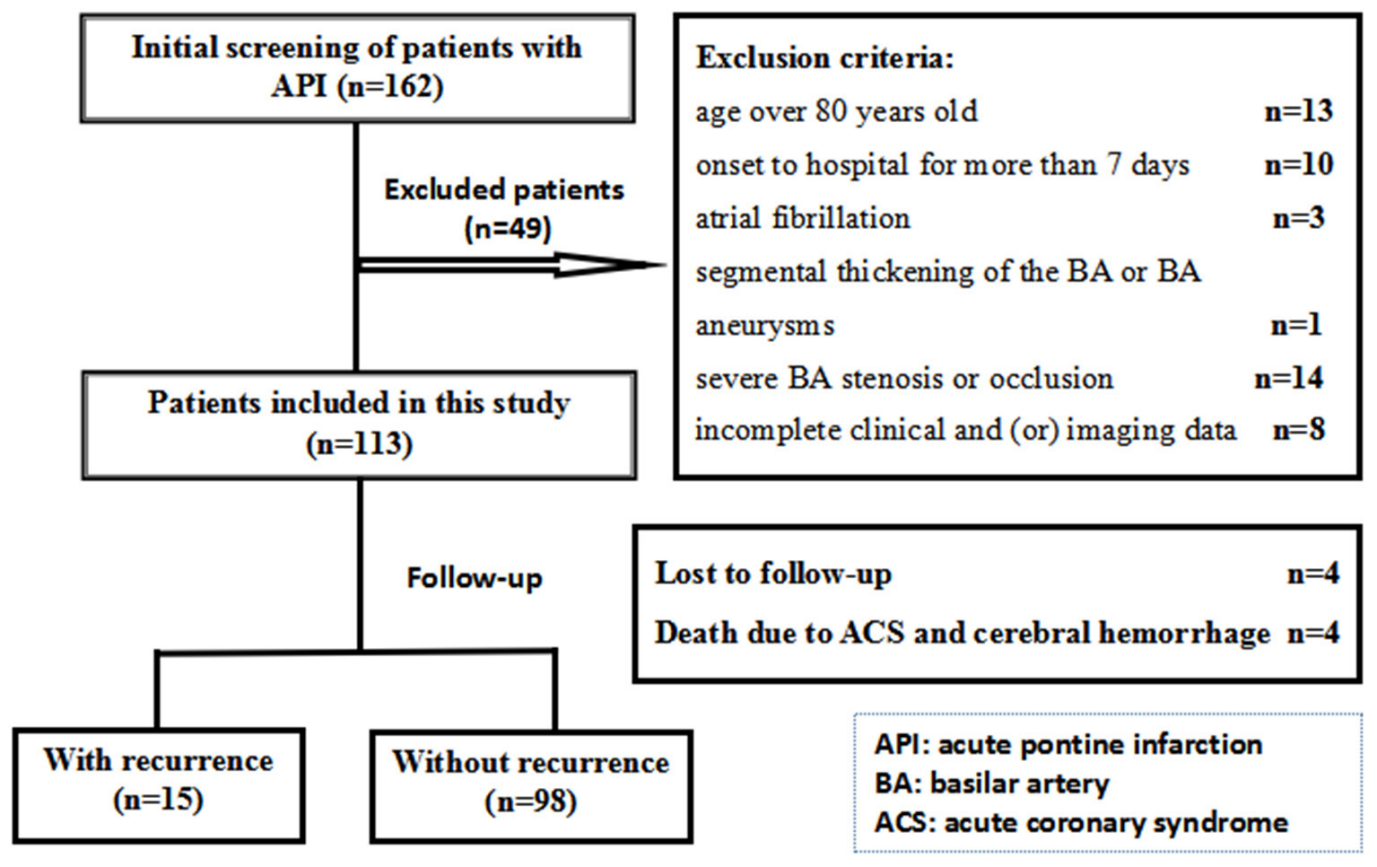
Study flowchart of the present study.

A total of 162 patients with API were identified for this study; 113 patients were ultimately included, as shown in Figure 1. The median age of onset was 64 years (IQR 56–73) years, and the sex ratio was 66:47 male: female. Among them, 39 (34.5%) patients were diagnosed with BA dolichosis, whereas the other 74 (65.5%) patients had no BA dolichosis. The mean diameters of BA were 2.89 ± 0.64 mm, with 3.13 ± 0.74 mm in patients with BA dolichosis and 2.76 ± 0.54 mm in patients without BA dolichosis. The 50^th^ percentile BA diameter was 2.90 mm. Only two patients were diagnosed with BA ectasia according the current criteria, and they were diagnosed with BADE due to the simultaneous presence of BA dolichosis. Sixty–six patients had BA curvature, including 64 cases of moderate curvature and 2 cases of prominent curvature (dolichosis). Among them, the maximum bend was at the distal portion of the BA in 3 cases, the middle of the BA in 61 cases, and the proximal portion of the BA in 2 cases. The proportion of BA curvature was significantly higher in patients with BA dolichosis than in those without BA dolichosis (82.1 vs. 45.9%, *P* < 0.001). The etiology of API included LAD in 25 patients (22.1%), BABD in 67 patients (59.3%), SAD in 8 patients (7.1%), and other and undetermined etiologies in 13 patients (11.5%). The clinical characteristics and imaging data of the BA are presented in Table 1.

**Table 1:** Clinical characteristics.

**Variables**	**Total (*n* = 113)**	**With BA dolichosis (*n* = 39)**	**Without BA dolichosis (*n* = 74)**	***P* value**
**Age (years)**	63.7 ± 10.0	64.1 ± 10.5	63.6 ± 9.9	0.799
**Age grouped by median**, ***n*** **(%)**				0.424
Age <65 y	58 (51.3)	18 (46.2)	40 (54.1)	
Age ≥65 y	55 (48.7)	21 (53.8)	34 (45.9)	
**Sex**, ***n*** **(%)**				0.373
Female	47 (41.6)	14 (35.9)	33 (44.6)	
Male	66 (58.4)	25 (64.1)	41 (55.4)	
**Risk factors**, ***n*** **(%)**				
Smoking	36 (31.9)	14 (35.9)	22 (29.7)	0.504
Drinking	18 (15.9)	9 (23.1)	9 (12.2)	0.132
Hypertension	94 (83.2)	36 (92.3)	58 (78.4)	0.060
Diabetes mellitus	49 (43.4)	10 (25.6)	39 (52.7)	0.006
Dyslipidemia	43 (38.4)	16 (42.1)	27 (36.5)	0.563
**Medications at discharge**, ***n*** **(%)**				
Antiplatelet agents				1.000
Single antiplatelet agents	99 (87.6)	34 (87.2)	65 (87.8)	
Dual antiplatelet agents	14 (12.4)	5 (12.8)	9 (12.2)	
Statins	113 (100)	39 (100)	74 (100)	–
Antihypertensive agents	84 (74.3)	35 (89.7)	49 (66.2)	0.006
Glucose-lowering agents	40 (35.4)	10 (25.6)	30 (40.5)	0.115
**Basilar artery features**, ***n*** **(%)**				
**BA curvature**				<0.001
No BA curvature	47 (41.6)	7 (17.9)	40 (54.1)	
Moderate curvature	64 (56.6)	30 (76.9)	34 (45.9)	
Prominent curvature	2 (1.8)	2 (5.1)	0	
**BA diameter (mm)**	2.89 ± 0.64	3.13 ± 0.74	2.76 ± 0.54	0.008
**BA diameter grouped by median**				0.087
<2.90 mm	56 (49.6)	15 (38.5)	41 (55.4)	
≥2.90 mm	57 (50.4)	24 (61.5)	33 (44.6)	
**BAH**	11 (9.7)	4 (10.3)	7 (9.5)	1.000
**BA stenosis**				0.196
No stenosis	70 (61.9)	24 (61.5)	46 (62.2)	
Mild stenosis	24 (21.2)	6 (15.4)	18 (24.3)	
Moderate stenosis	19 (16.8)	9 (23.1)	10 (13.5)	
**Location of pontine infarction**, ***n*** **(%)**				0.541
Deep pontine infarction	13 (11.5)	3 (6.7)	10 (13.5)	
Pontine base infarction	100 (88.5)	36 (92.3)	64 (86.5)	
**Stroke etiology**, ***n*** **(%)**				0.218
SAD	8 (7.1)	2 (5.1)	6 (8.1)	
BABD	67 (59.3)	23 (59.0)	44 (59.5)	
LAD	25 (22.1)	12 (30.8)	13 (17.6)	
Other and undetermined etiologies	13 (11.5)	2 (5.1)	11 (14.9)	

*BA, basilar artery; BAH, basilar artery hypoplasia; SAD, small-artery disease; BABD, basilar artery branch disease; VLAD, large-artery disease*.

**Figure 2:**
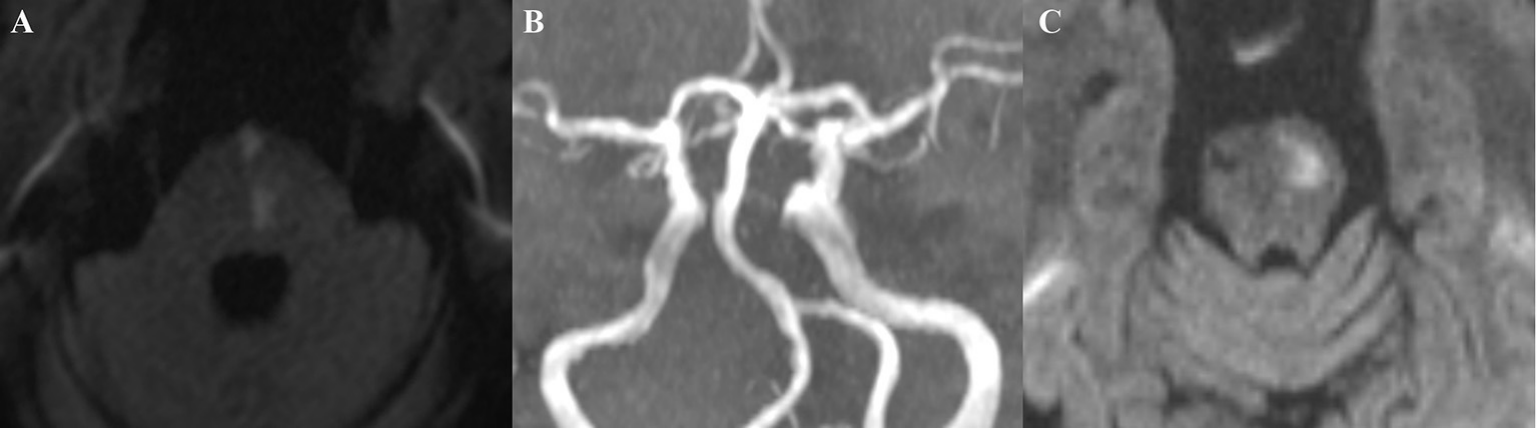
A typical case: This patient, a 69-year-old man with a history of hypertension and diabetes mellitus, was admitted to our hospital with right upper and lower extremity weakness for two days (Medical Research Council scale, grade IV). DWI showed left paramedian pontine infarction (middle pontine) **(A)**; MRA did not reveal basilar artery stenosis, while BA dolichosis and moderate curvature were noted **(B)**. The patient's muscle strength returned to normal after treatment. Four years later, the patient again developed weakness in the right upper and lower extremities, and a repeat DWI suggested the left basal infarction of upper pontine **(C)**.

The mean follow–up time was 31.2 months (range 0.9–59.7 months). At the last follow–up, three patients had stopped antiplatelet drugs due to adverse gastrointestinal reactions, one of whom had BA dolichosis, while two patients had no BA dolichosis. However, no patients discontinued statins. During follow–up, DWI–confirmed brainstem infarction occurred in 15 (13.3%) patients, with estimated 1–year, 3–year, and 5–year incidence rates of brainstem infarction recurrence of 2.7, 9.7, and 17.6%, respectively. Among these cases, one recurred in the medulla oblongata and cerebellum (this patient had no BA dolichosis), two in the midbrain and pons, and the other 12 in the pons (see Figure 2, a typical case), with the average time from onset to recurrence of 24.9 months (range 0.9–50.9 months). In patients with BA dolichosis, the recurrence rate reached 23.1%, which was significantly higher than the incidence of 8.1% in patients without BA dolichosis. During the whole follow–up period, four patients were lost to follow–up, for a loss to follow–up rate of 3.5%. At the end of follow–up, three patients had died of sudden acute coronary syndrome, and one patient had died of cerebral hemorrhage. None of the four patients had BA dolichosis. In addition, basal ganglia infarction recurred in three patients during the follow–up period, which was considered unrelated to BA dolichosis and was not regarded as a defined outcome event. Another patient had basal ganglia infarction first and then brainstem infarction. The recurrence time of brainstem infarction was taken as the follow–up end point. In addition, three patients had stroke symptoms during the follow–up, two of whom were considered to have transient ischemic attack (TIA), and one was diagnosed with DWI–negative cerebral infarction. However, it was uncertain whether it was brainstem infarction recurrence.

### Analysis of the Risk of Brainstem Infarction Recurrence

**Figure 3:**
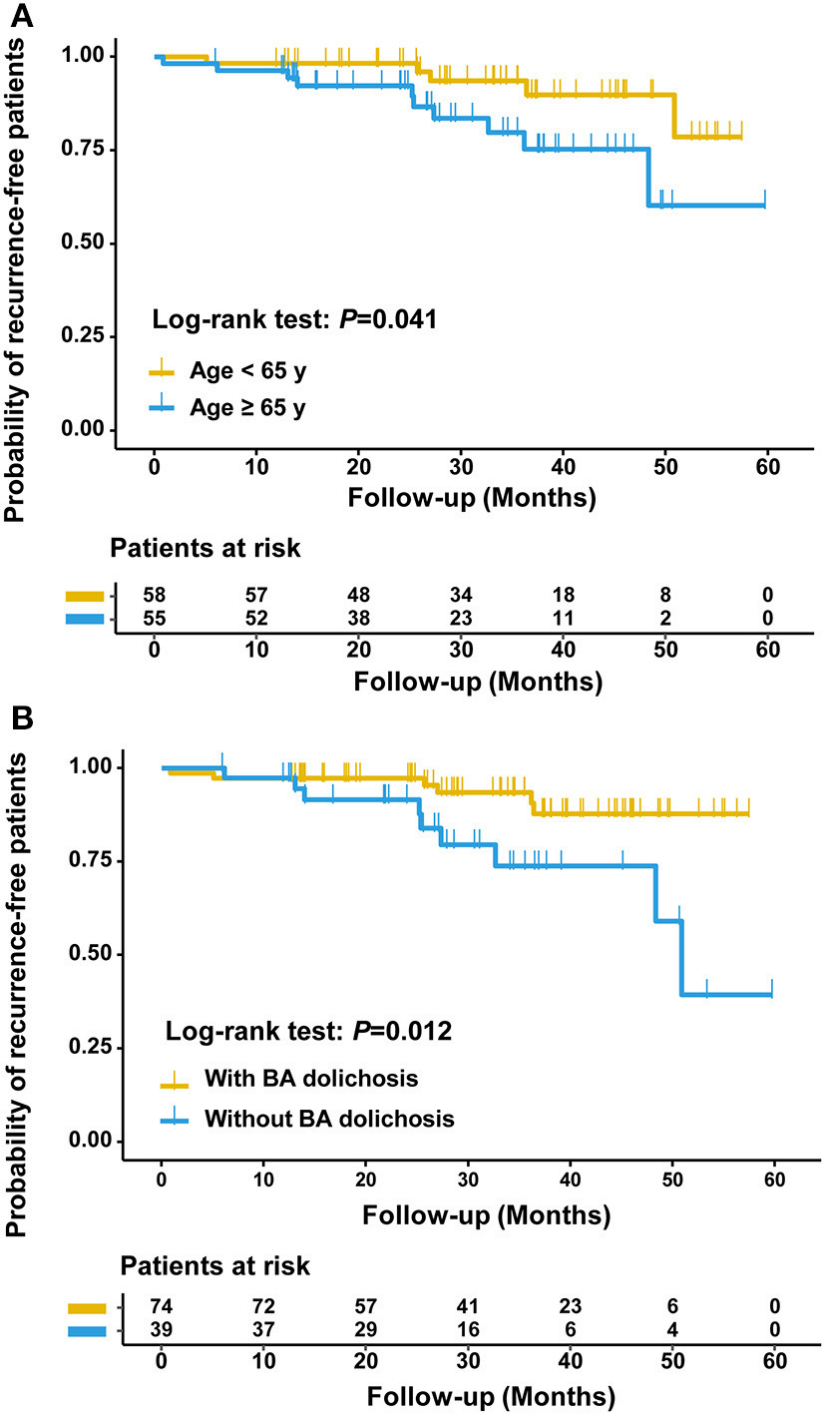
Kaplan-Meier curves estimating the probability of being recurrence-free between patients aged ≥65 years and aged <65 years **(A)** and between patients with and without BA dolichosis **(B)**.

Kaplan–Meier curve analysis revealed a significantly higher rate of brainstem infarction recurrence in the patients with BA dolichosis than in those without BA dolichosis (*P* = 0.012, log rank test). Age ≥65 years also significantly increased the risk of brainstem infarction recurrence (*P* = 0.041, log rank test) (Figure 3).

Univariate Cox proportional hazard analysis showed that BA dolichosis was associated with an increased risk of brainstem infarction recurrence (HR = 3.466, 95% CI: 1.229–9.779, *P* = 0.019). The risk of brainstem infarction recurrence was increased in patients with moderate curvature (HR = 2.057, *P* = 0.203) and prominent curvature (HR = 10.394, *P* = 0.036), using patients with no curvature as a reference. However, age, male sex, risk factors, medications at discharge, BA curvature, BA diameter, BAH, BA stenosis, location of pontine infarction and stroke etiology were not significantly associated with a higher risk of brainstem infarction recurrence. After adjusting for variables (*P* <0.15) with potential association, age ≥65 years (HR = 3.341, 95% CI: 1.079–10.348, *P* = 0.036), and BA dolichosis (HR = 3.048, 95% CI: 1.069–8.693, *P* = 0.037) were significantly associated with brainstem infarction recurrence (Table 2).

**Table 2:** Univariate and multivariate Cox proportional-hazards models for the risk of brainstem infarction recurrence.

**Variables**	**Univariate analysis**		**Multivariate analysis**	
	**HR (95%CI)**	***P value***	**HR (95%CI)**	***P* value**
**Age**	1.037 (0.983–1.093)	0.187		
**Age grouped by median**				
Age <65 y	Ref			
Age ≥65 y	3.011 (0.998–9.085)	0.050	3.341 (1.079–10.348)	0.036
**Sex**				
Female	Ref			
Male	2.802 (0.790–9.946)	0.111	3.564 (0.977–12.955)	0.054
**Risk factors**				
Smoking	1.483 (0.527–4.176)	0.456		
Drinking	1.258 (0.354–4.467)	0.723		
Hypertension	1.493 (0.336–6.642)	0.598		
Diabetes mellitus	0.932 (0.331–2.623)	0.894		
Dyslipidemia	0.982 (0.349–2.763)	0.973		
**Medications at discharge**				
Antiplatelet agents				
Single antiplatelet agents	Ref			
Dual antiplatelet agents	0.666 (0.087–5.103)	0.695		
Statins	–	–		
Antihypertensive agents	1.565 (0.438–5.593)	0.490		
Glucose-lowering agents	0.764 (0.242–2.414)	0.647		
**Basilar artery features**				
**BA dolichosis**	3.466 (1.229–9.779)	0.019	3.048 (1.069–8.693)	0.037
**BA curvature**				
No BA curvature	Ref			
Moderate curvature	2.057 (0.677–6.248)	0.203		
Prominent curvature	10.394 (1.159–93.239)	0.036		
**BA diameter (mm)**	1.347 (0.571–3.179)	0.496		
**BA diameter grouped by median**				
<2.90 mm	Ref			
≥2.90 mm	0.982 (0.355–2.715)	0.973		
**BAH**	0.498 (0.065–3.802)	0.501		
**BA stenosis**				
No stenosis	Ref			
Mild stenosis	2.188 (0.693–6.906)	0.182		
Moderate stenosis	1.952 (0.497–7.667)	0.338		
**Location of pontine infarction**				
Deep pontine infarction	Ref			
Pontine base infarction	1.486 (0.194–11.375)	0.703		
**Stroke etiology**				
SAD	Ref			
BABD	1.070 (0.119–9.627)	0.952		
LAD	1.076 (0.136–8.489)	0.945		
Other and undetermined etiologies	0	0.980		

*BA, basilar artery; BAH, basilar artery hypoplasia; SAD, small-artery disease; BABD, basilar artery branch disease; VLAD, large-artery disease*.

### Subgroup Analysis

Further analysis showed that age and sex both had interaction effects with BA dolichosis for the risk of brainstem infarction recurrence (*P* for trend < 0.001 and = 0.007, respectively). The subgroup analysis stratified by age demonstrated that BA dolichosis was highly associated with the risk of brainstem infarction recurrence in patients aged ≥65 years (HR = 7.319, 95% CI: 1.525–35.123, *P* = 0.013) (Table 3). We also carried out a subgroup analysis based on sex, but no association was found between BA dolichosis and brainstem infarction recurrence stratified by sex (Table 4).

**Table 3:** Subgroup analysis stratified by age.

**Variables**	**Age** **<** **65y**				**Age** **≧** **65 y**			
	**Total n**	**Events**	**HR (95%CI)**	***P* value**	**Total n**	**Events**	**HR (95%CI)**	***P* value**
With BA dolichosis	18	1	0.712 (0.079–6.408)	0.762	21	8	7.319 (1.525–35.123)	0.013
Without BA dolichosis	40	4	Ref	NA	34	2	Ref	NA

*BA, basilar artery; HR, Hazard ratio; CI, confidence interval; NA, not applicable*.

**Table 4:** Subgroup analysis stratified by sex.

**Variables**	**Female**				**Male**			
	**Total n**	**Events**	**HR (95%CI)**	***P* value**	**Total n**	***Events***	**HR (95%CI)**	***P* value**
With BA dolichosis	14	2	2.646 (0.161–43.464)	0.496	25	7	2.732 (0.845–8.827)	0.093
Without BA dolichosis	33	1	Ref	NA	41	5	Ref	NA

*BA, basilar artery; HR, Hazard ratio; CI, confidence interval; NA, not applicable*.

## Discussion

In this prospective cohort study, we found that age ≥65 years and BA dolichosis were significantly associated with brainstem infarction recurrence. In the subgroup analysis stratified by age, the patients aged ≥65 years with BA dolichosis had a higher risk of brainstem infarction recurrence. Therefore, the API patients with BA dolichosis may have a higher risk of recurrent brainstem infarction, especially in elderly patients.

BA dolichoectasia is a complex arteriopathy that can result in diverse neurologic complications, some of which may be more frequently explained by BA dolichosis and others by BA ectasia. In addition to common atherosclerotic factors, BA dolichosis or BA ectasia, which can lead to intraluminal thrombosis or perforating artery stenosis or occlusion through compression or stretching and subsequent hemodynamic abnormalities, may also increase the risk of ischemic stroke (14). The study by Passero et al. (15) focused on the long–term prognosis in patients with VBD. The end–point events they selected were relatively broad, such as ischemic stroke, without regard to whether the recurrent infarction was associated with VBD. However, VBD/BADE patients are at a high risk of posterior circulation stroke, with brainstem infarction being the most common (2–4). Therefore, we selected DWI–confirmed brainstem infarction as the endpoint event. In the Chinese population, BA ectasia is uncommon under the current criteria, while BA dolichosis is relatively more common (5). Whether BA dolichosis or BA ectasia is more likely to lead to posterior circulation infarction remains to be determined. In the present study, we demonstrated that BA dolichosis was associated with a higher risk of long–term brainstem infarction recurrence (HR = 3.048, P = 0.037). Although the BA diameter of patients with BA dolichosis was greater than that of patients without BA dolichosis, the BA diameter was not statistically significantly correlated with the recurrence of brainstem infarction. Additionally, no recurrence was noted in the two patients with BA ectasia at the end of the follow–up.

In the present study, patients with BA dolichosis were more likely to have BA curvature than those without BA dolichosis (82.1 vs. 45.9%). Our previous study also showed a positive correlation between BA curve length and BL (r = 0.550, *P* < 0.001) (5). Although BA curvature did not enter into the final multivariate Cox regression model possibly due to the relatively small sample size, the recurrence risk was 2.057 (*P* = 0.203) and 10.394 times (*P* = 0.036) higher in patients with moderate curvature and prominent curvature than in those with no curvature in the univariate Cox regression analysis. The study by Zhu et al. (16) showed that patients with BA curvature had a higher rate of posterior circulation infarction than those without BA curvature, and BA curvature led to a tendency for patients to develop posterior circulation infarction. Zhang et al. (17) also demonstrated that BL of level 3 (3.77–7.25 mm) was an independent risk factor for API. Moreover, the maximum bend was largely located at the middle of the BA in this study, which could also explain the fact that the infarct recurrence was mostly located in the pons.

Most studies show that BA dolichosis and curvature cause brainstem infarction due to hemodynamic abnormalities, *in situ* thrombosis, or perforating artery disease (5, 16, 17). First, BA dolichosis and curvature cause abnormal hemodynamics in the BA and atherosclerotic changes in the vessel wall, which aggravate the perforating artery disease. Second, perforating artery stretching due to BA curvature aggravates vasospasm and perforating artery disease, which are believed to increase the risk of ischemic stroke (8, 17). In addition, BA dolichosis or curvature often accompanies the asymmetrical development of vertebral arteries, and the associated asymmetrical flow aggravates BA dolichosis, curvature, or dilation, in turn further aggravating perforating artery disease and increasing the risk of long–term stroke recurrence (12, 16). Zhang et al. (17) found that BL was positively correlated with the differential diameter of the vertebral arteries, which along with exposure to vascular risk factors increased the risk of pontine infarction. BAL and BL measurements may be used to predict which patients are at higher risk of posterior circulation infarction. In short, BA dolichosis and/or curvature may be more closely related to infarction recurrence.

The association between BA dolichoectasia or recurrent cerebral infarction and older age is universally accepted (18, 19). This study showed that the risk of brainstem infarction recurrence was significantly higher in patients aged ≥65 years than in patients aged <65 years. Subgroup analysis showed that among older patients, the risk of recurrent brainstem infarction was significantly higher in the BA dolichosis group than in the non–BA dolichosis group, while an intergroup difference was not observed in younger patients, which also explains why most patients with BA dolichosis are asymptomatic when they are young but are susceptible to recurrent posterior circulation TIA and even posterior circulation infarction (especially brainstem infarction) when they become older. Therefore, elderly patients with BA dolichosis should be closely monitored for the risk of long–term ischemic stroke. Although males had a marginally higher risk of recurrent brainstem infarction (*P* = 0.054), subgroup analysis did not find a statistically significant difference in the risk of brainstem infarction recurrence between male and female patients with BA dolichosis. Deng et al. (6) showed that the mean BA diameter in men was 0.2 mm larger than that in women, indirectly indicating that the diameter of the BA may not be the main factor underlying recurrent brainstem infarction.

In this prospective cohort study, we used the semiquantitative criterion to define BA dolichosis, with DWI–confirmed brainstem infarction as the endpoint event, which provided robust support for the analysis of the relationship between BA dolichosis and the risk of long–term recurrence of brainstem infarction. Nevertheless, this study has some limitations. First, we only enrolled patients with API, a patient population with a high incidence of BA dolichosis and more severe perforating artery disease. As a result, the patients had a higher long–term risk of brainstem infarction recurrence than the general population. However, due to the time constraints of this study, the sample was relatively small, and few patients had an endpoint event. Second, DWI–confirmed brainstem infarction was set as the endpoint event. During the follow–up period, three patients with API had recurrent stroke symptoms (TIA and DWI–negative cerebral infarction), but we were unable to confirm whether the symptoms were attributed to recurrent brainstem infarction. In addition, some patients with recurrent brainstem infarction may not go to hospital because of mild or no symptoms. Therefore, this study may underestimate the risk of recurrent brainstem infarction. Third, we were unable to accurately evaluate the relationship between BA dolichosis and the extent of perforating artery disease due to a lack of effective methods to evaluate the extent of perforating artery disease of the BA. However, we believe that BA dolichosis or curvature will aggravate perforating artery disease. Last, we did not use high–resolution MRI to analyze the stability of the vessel wall or plaque. Negative arterial remodeling may contribute to underestimated arterial diameters, which may also explain the rarity of BA dilatation. Nevertheless, subgroup analysis showed no correlation between BA diameter and recurrent brainstem infarction.

## Conclusion

BA dolichosis may increase the risk of long–term brainstem infarction recurrence in patients with API, especially in elderly patients, and therefore warrants more attention in clinical practice.

## Data Availability Statement

The raw data supporting the conclusions of this article will be made available by the authors, without undue reservation.

## Ethics Statement

The studies involving human participants were reviewed and approved by the Institutional Review Board of the Second People's Hospital of Hefei. The patients/participants provided their written informed consent to participate in this study. Written informed consent was obtained from the individual(s) for the publication of any potentially identifiable images or data included in this article.

## Author Contributions

This work was conceptualized by MX, SC, and WX and all approved the protocol. Data collection was done by QW, XN, JH, PC, TG, SC, and JW. Statistical analysis was undertaken by XZ, XN, and SC. SC, XZ, and QW prepared the manuscript. MX and WX were recipients of the obtained funding and were involved in the interpretation of the data and the manuscript revision. All authors contributed to the article and approved the submitted version.

## Funding

This study was supported by grants from the Major Research Development Program of Anhui Province (1804h08020233) and the Major Applied Medical Research Foundation of the Health and Family Planning Commission of Hefei (hwk2017zd008).

## Conflict of Interest

The authors declare that the research was conducted in the absence of any commercial or financial relationships that could be construed as a potential conflict of interest.

## Publisher's Note

All claims expressed in this article are solely those of the authors and do not necessarily represent those of their affiliated organizations, or those of the publisher, the editors and the reviewers. Any product that may be evaluated in this article, or claim that may be made by its manufacturer, is not guaranteed or endorsed by the publisher.
